# Tubo-Ovarian Mass Leading to Necrotizing Fasciitis in a Patient With Systemic Lupus Erythematosus: A Case Report

**DOI:** 10.7759/cureus.80759

**Published:** 2025-03-18

**Authors:** Pooja Barekar, Varsha Kose, Prachi Dixit

**Affiliations:** 1 Department of Obstetrics and Gynecology, NKP Salve Institute of Medical Sciences and Research Centre and Lata Mangeshkar Hospital, Nagpur, IND; 2 Department of Obstetrics and Gynecology, All India Institute of Medical Sciences, Nagpur, IND

**Keywords:** autoimmune disease, lupus nephritis, necrotizing fasciitis, systemic lupus erythematosus, total abdominal hysterectomy

## Abstract

Acute abdominal pain is a common clinical problem in emergency and non-emergency cases. Acute abdomen in systemic lupus erythematosus (SLE) is a challenging diagnostic and therapeutic problem that carries high mortality and morbidity rates. The present study reports a case of a 45-year-old female, a known case of SLE, hypothyroidism, and seizure disorder. The patient presented with chief complaints of pain in the left iliac and lumbar region with a history of amenorrhea for three to four months. Based on the clinical findings, laboratory investigations, and diagnostic assessment involving ultrasonography (USG), the diagnosis of acute abdomen along with tubo-ovarian mass with dermoid cyst and SLE with lupus nephritis and seizure disorder was confirmed. The patient was operated with total abdominal hysterectomy (TAH) with bilateral salpingo-oophorectomy with loop colostomy. The patient was placed in the surgical intensive care unit for observation; however, sepsis developed, and the trauma from the exploratory laparotomy led to septic shock, followed by hypotension and ultimately cardiac arrest. Hence, the cause of death was attributed to sepsis with shock and multiple organ dysfunction syndromes. In conclusion, acute abdomen in SLE suggests that systemic measurement and early laparotomy may improve the prognosis; however, due to chronic inflammatory status and immune-compromised state, the cases should be handled with utmost caution with a multidisciplinary approach due to the increase in the mortality rate.

## Introduction

Systemic lupus erythematosus (SLE) is an autoimmune disease consisting of various immunological abnormalities and clinical presentations; however, the etiology is still unknown with dominance in females in comparison to males demonstrating a ratio of 9:1 [1,2]. The clinical features most commonly highlighted consist of musculoskeletal, cutaneous, cardiovascular, respiratory, hematological, renal, and neuropsychiatry systems along with constitutional symptoms involving weight loss, fatigue, and fever [3,4]. Lupus nephritis with immune complex deposition can affect all four components involving vessels, tubules, interstitium, and glomeruli [5]. The selection of treatment is determined based on the affected organ system(s) and the degree of involvement, varying from basic interventions (antimalarials, non-steroidal anti-inflammatory drugs {NSAIDs}) to intensive therapies (corticosteroids, cytotoxic agents) [6]. Furthermore, SLE demonstrated an elevated risk of Hodgkin lymphoma [7], as well as malignancies of the thyroid, liver, lung, and vagina [8,9].

Moreover, for emergency care clinicians, abdominal pain poses diagnostic challenges. In several instances, differential diagnosis varies significantly from benign to life-threatening diseases for which the causes include intra- and extra-abdominal conditions and other medical and surgical ailments. Atypical presentations of common diseases appear frequently and associated symptoms lack specificity, leading to diagnostic and therapeutic challenges [10]. Hence, this study highlights a case of SLE presenting as acute abdomen with left tubo ovarian mass.

## Case presentation

Patient information

A 45-year-old female presented to the Department of Obstetrics and Gynecology with chief complaints of pain in the left iliac and lumbar regions for one month, which had increased severely over the past two days. The patient reported a history of SLE along with seizure disorder for 20 years and hypothyroidism for one year. The patient has been prescribed 10 mg prednisolone for SLE, a tablet of sodium valproate 200 mg for seizure episodes, and a tablet levothyroxine 75 mcg for hypothyroidism. The patient reported a history of amenorrhea for three to four months, and the patient had similar complaints of amenorrhea 1.5 years back followed by bleeding. The patient has had one pregnancy, resulting in one living child, delivered by cesarean section at 37 weeks due to severe pre-eclampsia.

Additionally, the patient had a turbulent course during pregnancy and was on treatment. The last childbirth was 18 years ago. The patient was allergic to antibiotics such as non-steroidal anti-inflammatory drugs (NSAIDs), codeine, and methotrexate.

Clinical examination

On physical examination, the patient was vitally stable and the thyroid was not palpable demonstrating that it was not enlarged or not easily felt upon palpation reporting a normal finding. However, tenderness was present in the left iliac fossa and hypogastrium region. On per speculum examination, the cervix and vagina were found to be healthy. On per vaginal examination, the uterus was of normal size and retroverted. Bilateral fornix was free and tenderness was present on the left fornix.

The laboratory investigations reported that the hemoglobin level was 11 g/dL suggesting mild anemia, the white blood cell count was 22,600 cells/cumm indicating elevated white blood cell count, and the platelet count was 4.92 lakh/cumm which was also found to be slightly elevated. The erythrocyte sedimentation rate (ESR) was 20 mm/h suggesting elevated ESR indicating the presence of SLE, and the C-reactive protein (CRP) was 28 mg/dL indicating inflammation in the body. The thyroid-stimulating hormone (TSH) value was 10 µIU/mL, free triiodothyronine (FT3) was 13.29 pmol/L, and free thyroxine (FT4) was 2.65 pmol/L indicating hypothyroidism. The per speculum examination revealed cervical erosion.

Diagnostic assessment

**Figure 1:**
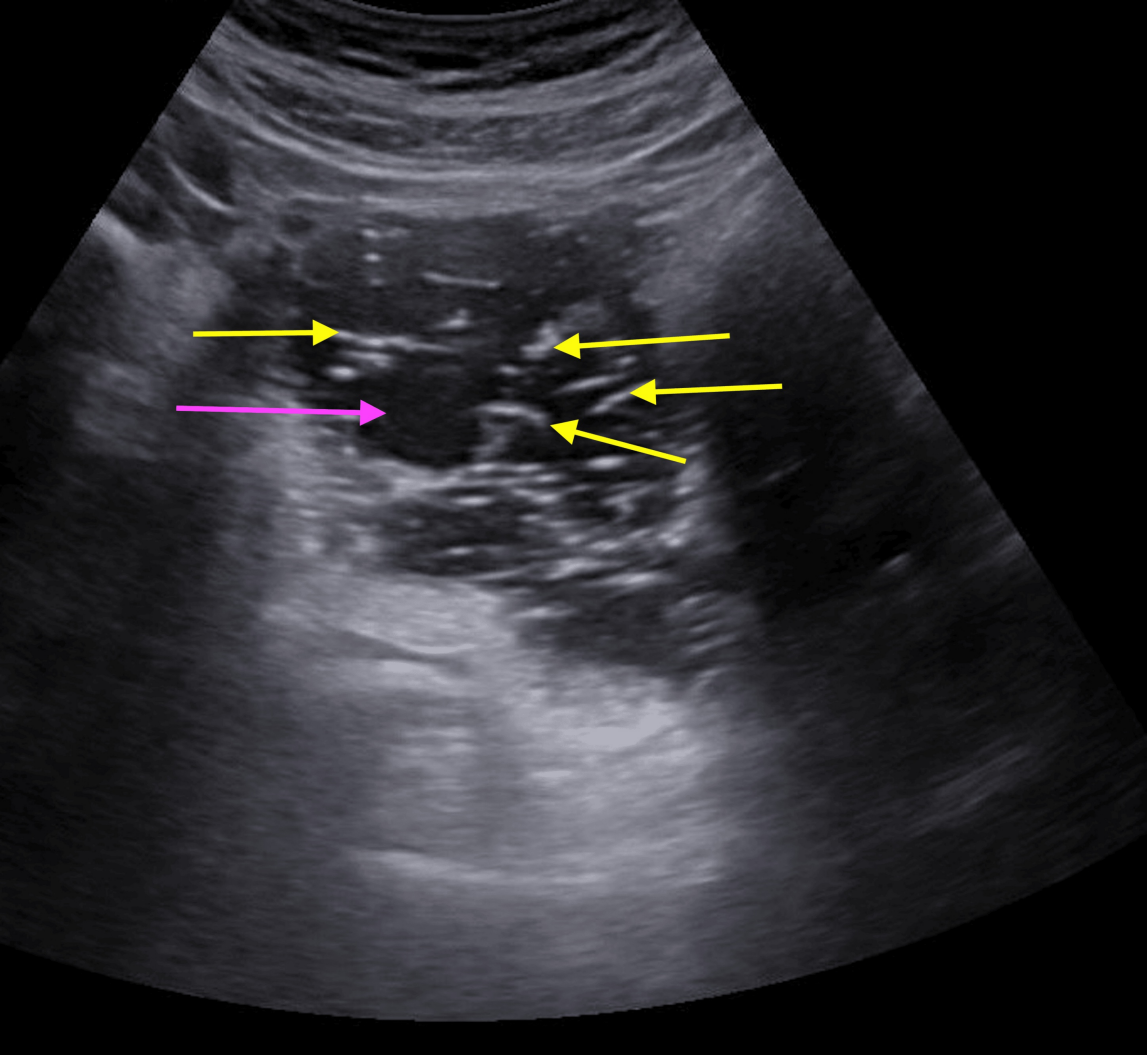
Pelvic ultrasonography (USG). The pink arrow indicates a hypoechoic lesion measuring 8.2 x 3.7 cm in the left adnexa, while the yellow arrows highlight multiple hyperechoic foci within the lesion, appearing as thin linear structures resembling hair strands.

On abdominal and sagittal transvaginal ultrasonography (USG), anterior and posterior myometrium appeared slightly heterogeneous, and endometrial thickness was 5 mm. A well-defined heterogeneous predominantly hypoechoic lesion of size 8.2 x 3.7 cm was noted in the left adnexa. Moreover, few hyperechoic foci were noted within the lesion. Multiple hyperechoic thin linear structures were observed that were suggestive of hair strands. Additionally, minimal peripheral vascularity was noted. The lesion was likely arising from the left ovary. Hence, the USG report indicated a dermoid cyst of the left ovary with early changes of adenomyosis as illustrated in Figure 1. Based on the above-mentioned investigations, the clinical diagnosis of acute abdomen with left tubo-ovarian mass and dermoid cyst, along with SLE, lupus nephritis, hypothyroidism, and seizure disorder, was confirmed.

Therapeutic intervention

**Figure 2:**
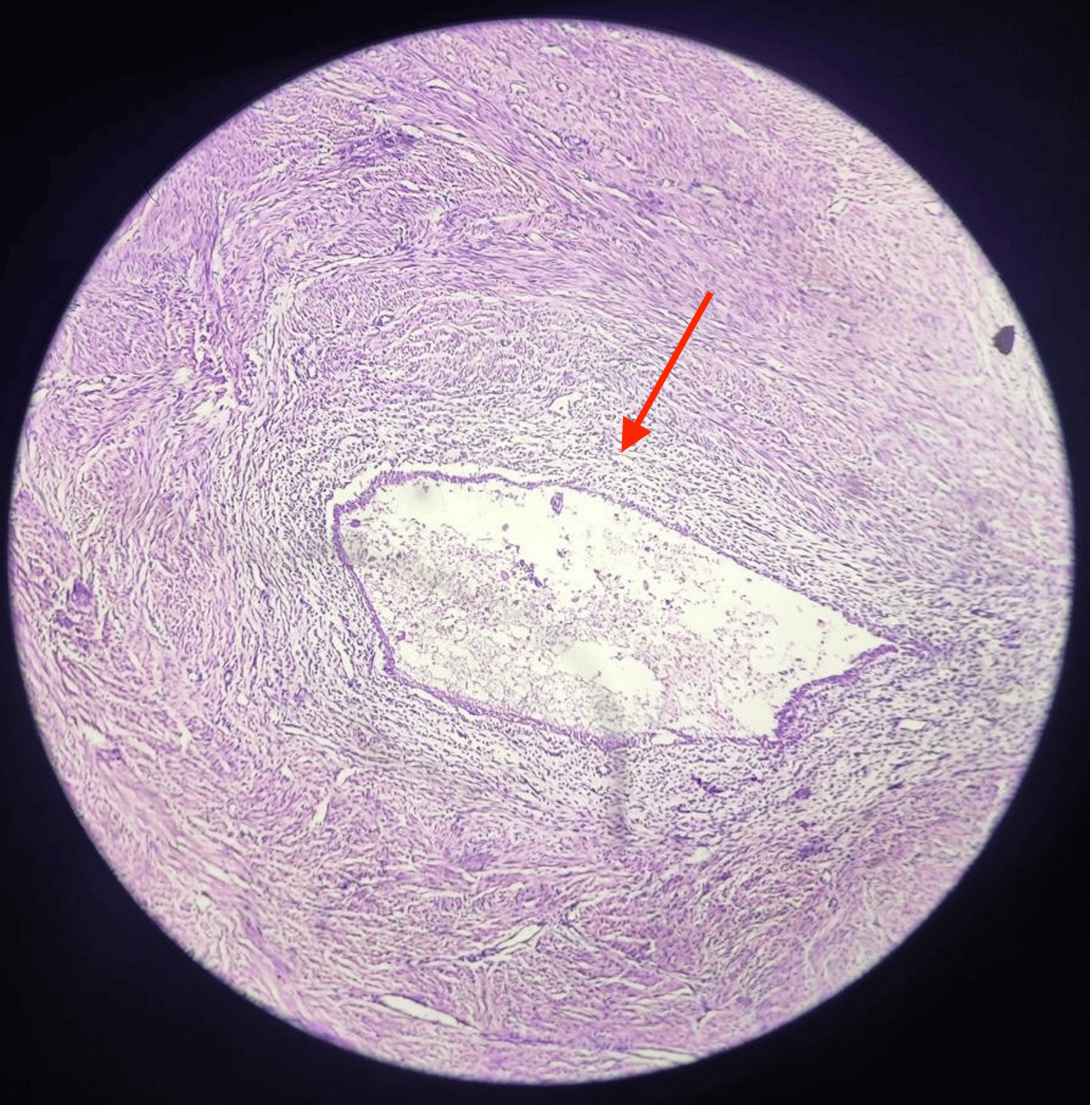
Histopathological examination illustrating adenomyoma in myometrium. ⁠The image shows the adenomyosis inside a leiomyoma (adenomyoma) in myometrium (red arrow).

The patient was planned for exploratory laparotomy with excision of tubo ovarian cyst which got converted to total abdominal hysterectomy (TAH) with bilateral salpingo-oophorectomy, and loop colostomy as adhesion was noted between posterior wall of uterus and sigmoid colon. The intra-operative dense adhesion was present between the posterior wall of the left tube, ovary, uterus, and sigmoid colon. During the separation of adhesion, an iatrogenic defect of the injured bowel of 0.5 cm was noted. Primary closure of defects was performed. Proximal diversion of the colon with colostomy on the left side was done. An intra-abdominal drain was placed in situ. The specimens were sent for histopathological examination (HPE), and the reports suggested cervix showing chronic cervicitis, adnexal mass showing a unilocular cyst containing cheesy and sebaceous grey-white material with a wrinkled epidermis-like wall from a hair shaft protruding into the cyst, and the endometrium showing simple glandular hyperplasia and myometrium showing adenomyoma, as illustrated in Figure 2.

After four post-operative days, anterior abdominal wall muscle necrosis was observed at the incision site. The non-fermenter bacteria was responsible for the pus and approximately 100 mL of pus was collected at the stump of the hysterectomy, aspirated, and the necrosed tissue was excised. Ceftriaxone which is a third-generation cephalosporin was used in the initial surgery and piperacillin/tazobactam was selected based on the balance of bacteria in the abscess. The strategy for the use of antimicrobial agents, when sepsis occurred, was to ensure a positive outcome and to reduce adverse antibiotic effects as well as prevent drug resistance. The colostomy was refreshed with vacuum-assisted closure (VAC) dressing, and an abdominal drain (ADK drain no. 32) was kept in situ.

However, necrotizing fasciitis was observed over the anterior abdominal wall for which the devitalized tissue was excised. The splenic flexure and hepatic flexure were mobilized and ADK drains no. 32 were kept in situ. A colocolonic anastomosis was performed. Two ROMOVAC drains no. 42 were kept in the subcutaneous plain and anatomical repair with transverse colostomy and end colostomy closure with VAC dressing was done. Because of necrotizing fasciitis of the anterior abdominal wall, likely due to gas gangrene, the patient required hyperbaric oxygen therapy, O_2_ support, and incentive spirometry. However, worsening of the wound site along with discoloration and erythema was observed. The patient landed into septic shock for which intubation and hyperbaric oxygen therapy were given. The patient was kept on noradrenalin which was given to treat low blood pressure. However, the condition of the patient worsened. The patient’s condition deteriorated significantly, with non-recordable pulse and blood pressure, indicating severe cardiovascular instability and the electrocardiogram (ECG) showed a flatline, indicating cardiac arrest. Hence, the cause of death was attributed to sepsis with shock and multiple organ dysfunction syndromes, resulting from the complications of TAH and bilateral salpingo-oophorectomy with an iatrogenic bowel injury.

## Discussion

SLE is a chronic multisystemic autoimmune disease [11]. SLE patients who appear with an acute abdomen provide a special diagnostic challenge for surgeons since the differential diagnosis is wide and sometimes challenging to establish [12]. Acute abdomen is a serious and sometimes fatal condition that has to be diagnosed and treated early in SLE. Numerous studies have shown that in 60% of SLE patients, acute abdomen is caused by vasculitis [12]. Necrosis, intestinal ischemia, cholecystitis, and pancreatitis, are among the conditions that cause acute abdomen in SLE patients consequently, requiring surgical intervention. Additionally, those with active SLE may sometimes have reported acute abdomen unrelated to the disease that necessitates surgery [12]. Hence, the outcome of the patient depends on the pre-existing condition.

In the present case, clinical diagnosis of acute abdomen with left tubo ovarian mass and dermoid cyst with SLE, lupus nephritis, hypothyroidism, and seizure disorder was confirmed. Comparably, a study of the literature identifies two European case reports of two SLE-afflicted female patients in their 20s who first presented with stomach problems, which turned out to be appendicitis as a result of autoimmune vasculitis [13,14]. Additionally, Fukatsu et al. presented a case of an elderly Japanese woman with SLE who presented with the symptoms of occasional epigastric pain and intermittent abdominal pain [10]. In comparison, the current case reported an additional tubo-ovarian mass, necessitating surgical intervention. Furthermore, necrotizing fasciitis, as observed in this case, is a rare but severe complication, possibly aggravated by the patient’s immunocompromised status due to both the underlying SLE and surgical intervention [14].

SLE patients are prone to an increased risk of malignancies and gynecological conditions [8,9]. However, the combination of tubo-ovarian mass, lupus nephritis, and hypothyroidism leading to severe complications such as necrotizing fasciitis post-surgery is rare. The patient’s course of treatment, including exploratory laparotomy, TAH, and subsequent complications such as septic shock, represents a cascade of events that may be linked to the immunosuppressive nature of SLE and the accompanying long-term use of immunosuppressant and corticosteroids [5,8].

The presence of adenomyosis, chronic cervicitis, and necrotizing fasciitis in the HPE report of the dermoid cyst was found to be consistent with previous reports of gynecological complications in SLE patients [9]. The progression towards septic shock and multiple organ dysfunction underscores the complexity of managing SLE patients with surgical and post-surgical complications as prolonged lymphopenia may be a biomarker for immunosuppression caused by sepsis and can predict both early and late mortality rates [15].

## Conclusions

This study illustrates the challenges encountered in diagnosing and managing SLE patients with acute abdominal presentations and tubo-ovarian masses. It underscores the importance of careful pre-operative evaluation and post-operative care in such patients, given their susceptibility to severe complications like necrotizing fasciitis and septic shock. Surgical intervention, while necessary, may lead to rapid deterioration due to the immunosuppressive nature of the disease and its treatments. Therefore, accurate history physical examination, and surgical data should be reviewed before planning any intervention. Due to chronic inflammatory status and immune-compromised state, the case should be handled with utmost caution with a multidisciplinary approach, and proper counseling of the patient and family is very important because morbidity and sometimes mortality can also be faced. Hence, early recognition and aggressive management of post-surgical infections are crucial to improving outcomes in SLE patients.
